# Comparative evaluation of Savanna HSV 1+2/VZV multiplex assay and Simplexa HSV 1&2 and VZV direct kits for rapid molecular detection of HSV-1, HSV-2, and VZV

**DOI:** 10.1128/spectrum.03654-25

**Published:** 2026-02-04

**Authors:** Blake W. Buchan, Paul A. Granato, Jessica S. Hoff, Marie Wisniewski, Adam Nielsen, Lavannya Sabharwal, Derek Gerstbrein, Amorina Purpora, Puspa Bhattarai, Julie O'Neill, Kevin Knect, Cecelia Plummer, Preeti Pancholi

**Affiliations:** 1 Medical College of Wisconsin 5506, Milwaukee, Wisconsin, USA; 2 Laboratory Alliance of Central New York 51972 , Liverpool, New York, USA; 3 The Ohio State University Wexner Medical Center 12306, Columbus, Ohio, USA; Indiana University School of Medicine, Indianapolis, Indiana, USA

**Keywords:** herpes simplex virus, varicella-zoster virus, multiplex assay

## Abstract

Herpes simplex virus (HSV-1 and HSV-2) and varicella-zoster virus (VZV) cause cutaneous and mucocutaneous lesions. Primary infection and secondary reactivations affect the mouth, genitalia, and dermis at other bodily regions. Accurate clinical diagnosis without laboratory confirmation can be difficult since lesions caused by these viruses are nondescript, and areas of bodily infection can overlap. Rapid molecular test results have been shown to reduce the time to definitive diagnosis, providing additional opportunities for patient counseling, antiviral therapy, and appropriate infection prevention measures. A molecular, multiplexed HSV + VZV assay allows both viruses to be tested simultaneously from a single lesion swab. This could potentially enhance diagnostic accuracy through laboratory confirmation, thus minimizing dependence on clinical judgment alone. The recently Food and Drug Administration (FDA)-approved Savanna HSV 1+2/VZV multiplex assay has not been widely evaluated against other FDA-approved assays. This is the first report evaluating the clinical performance of the Savanna HSV 1+2/VZV assay compared to independent assays for HSV and VZV. Results indicated that the Savanna HSV 1+2/VZV assay was comparable to the Diasorin Simplexa HSV 1&2 Direct and VZV Direct assays.

**IMPORTANCE:**

Lesion-causing viruses, herpes simplex (HSV-1 and HSV-2) and varicella-zoster virus (VZV), are common and difficult to distinguish clinically. Each can be found over any area of the body but have different treatment regimens and considerations for infectivity and future outbreak management. Laboratory analysis by molecular assay is the gold standard for these viruses, but it can take hours to days and is reliant on the clinician choosing the correct virus to test the first time, or risk being outside the treatment window. A rapid molecular assay that detects all three viruses simultaneously was developed and recently approved by the Food and Drug Administration (FDA). Here, we provide data highlighting the clinical performance of the Savanna HSV 1+2/VZV assay and its ability to detect across anatomical locations and ages. These data indicate that the Savanna assay is comparable to individual assays for each viral target, providing the opportunity for enhanced diagnostic accuracy of lesions.

## INTRODUCTION

Herpes simplex virus (serotypes HSV-1 and HSV-2) and varicella-zoster virus (VZV) are members of the Herpesviridae family of DNA viruses. These viruses cause nondescript cutaneous and mucocutaneous lesions that can occur over the mouth, genitalia, and other body regions. HSV and VZV lesions can follow primary infection by the virus or result from reactivation of the latent virus causing recurrent episodes of the disease. Each virus has individual recommendations for proper epidemiological control measures and treatment (1, 2); thus, differentiation between HSV-1, HSV-2, and VZV is necessary for improved outcomes and limiting transmission.

Although both serotypes of HSV have been shown to cause disease in all locations of the body, HSV-2 is more frequently associated with genital infections via venereal transmission, while HSV-1 is more commonly associated with oral lesions (cold sores) (3, 4). Despite these historic trends, recent studies have highlighted an increasing prevalence of genital HSV-1 and oral HSV-2 infections, thereby making differentiation based on clinical and epidemiologic factors less reliable (3, 5, 6). Genital herpes affects approximately 27% of adults in the U.S., and up to 80% of adults have serologic evidence of oral herpes caused by HSV-1 (3, 4). Severity of outbreaks, duration of latency, and subsequent reactivations of HSV may differ based on serotype, location, and immune status of the patient (2). Secondary complications include HSV encephalitis and blindness in the cases of ocular HSV (2). Primary VZV infection (chickenpox) manifests as a pruritic, vesicular rash over the entire body. Reactivation (shingles) most commonly appears as painful lesions restricted to a single dermatome; however, lesions may also be less conspicuous and appear as oral, nasal, genital, or atypical dermal lesions.

A clinical validation study of the recently Food and Drug Administration (FDA)-approved sample-to-answer Savanna HSV 1+2/VZV (QuidelOrtho) multiplexed assay has recently been published; however, the publication only compared the Savanna assay to other multiplex assays that are infrequently used in clinical laboratories (7). This study evaluates the performance of the Savanna HSV-VZV assay compared to the Diasorin assays on clinical samples from multiple U.S. testing sites.

## RESULTS

### Patient demographic characteristics

The population included patients presenting with cutaneous and/or mucocutaneous oral, genital, or cutaneous (non-oral and non-genital) lesions. There were 44.1% male (215 of 488) and 55.9% female (273 of 488) patients for cutaneous lesion collections and 27.6% male (139 of 503) and 72.4% female patients (364 of 503) for mucocutaneous lesion collections (Table S1). An additional 16 specimens were not categorized as either cutaneous or mucocutaneous or were categorized as a combination mucocutaneous and cutaneous samples. These samples were included in the total section of Table S1. The median age for the entire study was 43 years (range = birth to 95 years).

### Clinical performance of Savanna HSV 1+2/VZV

Specimens with a clinical order for HSV (*n* = 635) or VZV (*n* = 374) were analyzed with the Savanna HSV 1+2/VZV assay and compared to the institutional test of record for three study sites, Simplexa HSV 1&2 and Simplexa VZV (Table 1). Discrepant results were adjudicated using a third molecular result performed by the Lyra or Solana assay. The positive and negative percent agreements of the Savanna HSV 1+2/VZV assay for a total of 635 samples across all age groups tested for HSV-1 were 98.9 and 98.7%, respectively (Table 1). Using the same comparison parameters, the PPA and NPA of the Savanna HSV 1+2/VZV assay for a total of 635 samples across all age groups tested for HSV-2 were 100.0 and 99.8%, respectively (Table 1). Likewise, the PPA and NPA of the Savanna HSV 1+2/VZV assay for a total of 374 samples across all age groups tested for VZV were 100.0 and 99.6%, respectively (Table 1). Based on *κ*, these values were categorized as almost perfect (>0.90).

**TABLE 1:** Savanna HSV 1+2/VZV assay clinical performance of all samples compared with Diasorin Simplexa HSV 1&2 Direct and VZV Direct testing and discrepant analysis by Lyra Direct HSV 1+2/VZV and Solana HSV 1+2/VZV^*a*^

Analyte	Total	TP	FN	TN	FP	OPA%	PPA %	NPA %	PPV %	NPV %
							95% CI	95% CI	95% CI	95% CI
HSV-1	635	185	2	442	6	98.70%	98.9% (185/187)	98.7% (442/448)	96.9% (185/191)	99.5% (442/444)
							89.03 to 96.46%	97.04 to 99.49%	93.30 to 98.56%	95.15 to 98.25%
HSV-2	635	157	0	477	1	99.80%	100.0% (157/157)	99.8% (477/478)	99.4% (157/158)	100.0% (477/477)
							91.40 to 98.27%	98.82 to 99.99%	95.68 to 99.91%	97.02 to 99.28%
VZV	374	126	0	247	1	99.70%	100.0% (126/126)	99.6% (247/248)	99.2% (126/127)	100.0% (247/247)
							93.35 to 99.52%	97.75 to 99.99%	94.69 to 99.89%	96.37 to 99.60%

^*a*^ CI, confidence interval; FN, false negative; FP, false positive; HSV-1, herpes simplex virus 1; HSV-2, herpes simplex virus 2; NPA, negative percent agreement; NPV, negative predictive value; OPA, overall percent agreement; PPA, positive percent agreement; PPV, positive predictive value; TN, true negative; TP, true positive; VZV, varicella-zoster virus.

**Fig 1:**
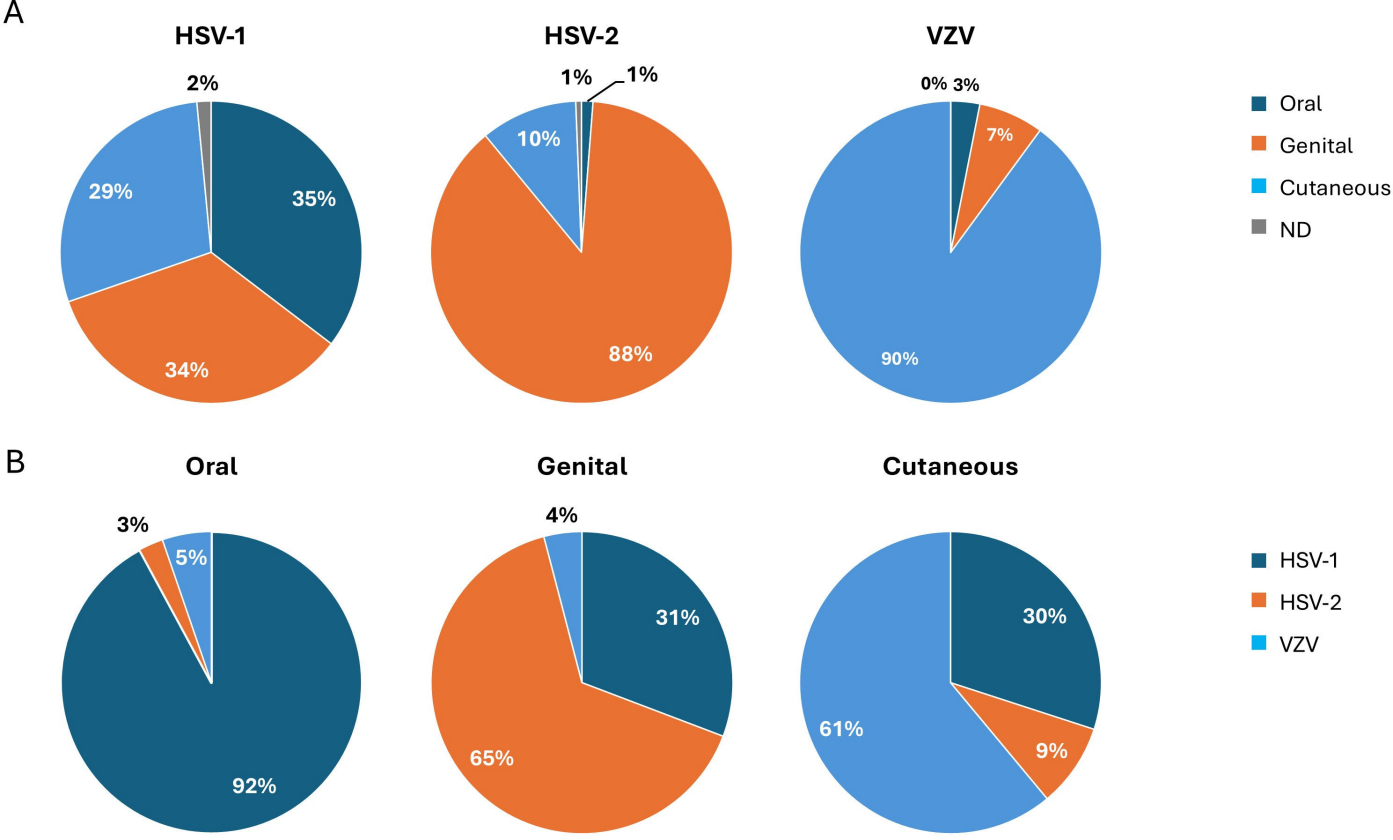
Viral positivity was categorized by anatomic site of sampled lesion. Panel **A** depicts the distribution of lesion sites reported as positive for each virus. Panel **B** depicts the distribution of viral positivity at each lesion site. ND, specimen source not defined by clinic.

Viral positivity was categorized by anatomic site of the sampled lesion (Fig. 1). Surprisingly, an equal prevalence of HSV-1-positive lesions was found in the genital region (34%) as opposed to the oral (35%) and other areas of the body (29%). Most HSV-2 lesions were identified in the genital area (88%), as expected with a sexually transmitted virus, and VZV lesions were detected mostly outside the oral and genital areas of the body (90%), although genital VZV was detected in 7% of cases.

A comparison of positive and negative results between the Savanna HSV 1+2/VZV assay and Diasorin Simplexa HSV 1&2 Direct and VZV Direct testing is summarized in Table S2 with discrepant analysis in Tables S3 to S5. There were seven, one, and one unresolved discrepancies between the tests for HSV-1, HSV-2, and VZV, respectively.

Robustness of the test comparison between Savanna and Simplexa was not affected by different lesion sites (Table 2), with positive percent agreement (PPA), negative percent agreement (NPA), PPV, and NPV all above 90%, most percentages at or approaching 100%. The number of true positive oral samples was highest for HSV-1, while HSV-2 had the highest number of true positives for genital samples compared to other pathogens. Oral and genital lesion sites had higher percentages of HSV-1 and HSV-2 than VZV positives, as expected. In cutaneous sites, HSV-1 and HSV-2 had a combined total of 70 true positive samples, while VZV had 113 (Table 2). Three samples were submitted from the clinic as pooled oral and genital samples and tested negative for all analytes on both platforms. A comparison of positive Ct values between Savanna and Simplexa is given in Table S6. The difference between Ct values reported by Simplexa vs. Savanna was significant, as indicated by *P*-values of 0.0001 for HSV-1 and <0.0001 for HSV-2 and VZV.

**TABLE 2:** Savanna HSV 1+2/VZV assay clinical performance of all samples by specimen source compared with Diasorin Simplexa HSV 1&2 Direct and VZV Direct testing and discrepant analysis by Lyra Direct HSV 1+2/VZV and Solana HSV 1+2/VZV^*a*^

		Total	TP	FN	TN	FP	PPA %	NPA %	PPV %	NPV %
							95% CI	95% CI	95% CI	95% CI
Oral	HSV-1	154	65	2	85	2	97.00%	97.70%	97.00%	97.70%
							89.63 to 99.64%	91.94 to 99.72%	89.19 to 99.22%	91.56 to 99.40%
	HSV-2	154	1	0	153	0	100.00%	100.00%	100.00%	100.00%
							2.50 to 100.00%^*b*^	97.62 to 100.00%	2.50 to 100.00%^*b*^	97.62 to 100.00%
	VZV	36	4	0	32	0	100.00%	100.00%	100.00%	100.00%
							39.76 to 100.00%^*b*^	89.11 to 100.00%	39.76 to 100.00%^*b*^	89.11 to 100.00%
Genital	HSV-1	307	63	0	240	4	100.00%	98.40%	94.00%	100.00%
							94.31 to 100.00%	95.86 to 99.55%	85.63 to 97.65%	98.47 to 100.00%
	HSV-2	307	139	0	167	1	100.00%	99.40%	99.30%	100.00%
							97.38 to 100.00%	96.73 to 99.98%	95.17 to 99.90%	97.82 to 100.00%
	VZV	74	9	0	65	0	100.00%	100.00%	100.00%	100.00%
							66.37 to 100.00%	94.48 to 100.00%	66.37 to 100.00%	94.48 to 100.00%
Cutaneous	HSV-1	166	54	0	112	0	100.00%	100.00%	100.00%	100.00%
							93.40 to 100.00%	96.76 to 100.00%	93.40 to 100.00%	96.76 to 100.00%
	HSV-2	166	16	0	150	0	100.00%	100.00%	100.00%	100.00%
							79.41 to 100.00%	97.57 to 100.00%	79.41 to 100.00%	97.57 to 100.00%
	VZV	260	113	0	146	1	100.00%	99.30%	99.10%	100.00%
							96.79 to 100.00%	97.51 to 100.00%	96.79 to 100.00%	97.51 to 100.00%
ND	HSV-1	5	3	0	2	0	100.00%	100.00%	100.00%	100.00%
							29.24 to 100.00%^*b*^	15.81 to 100.00%^*b*^	29.24 to 100.00%^*b*^	15.81 to 100.00%^*b*^
	HSV-2	5	1	0	4	0	100.00%	100.00%	100.00%	100.00%
							2.50 to 100.00%^*b*^	39.76 to 100.00%^*b*^	2.50 to 100.00%^*b*^	39.76 to 100.00%^*b*^
	VZV	1	0	0	1	0	N/A	100.00%	N/A	100.00%
								2.50 to 100.00%^*b*^		2.50 to 100.00%^*b*^

^*a*^ CI, confidence interval; FN, false negative; FP, false positive; HSV-1, herpes simplex virus 1; HSV-2, herpes simplex virus 2; ND, specimen source not defined by clinic; N/A, not available due to small sample size; PPA, positive percent agreement; NPA, negative percent agreement; NPV, negative predictive value; PPV, positive predictive value; TN, true negative; TP, true positive; VZV, varicella-zoster virus.

^*b*^ Low sample size contributes to variation CI range.

Distribution of individuals positive for HSV-1, HSV-2, and VZV was categorized by age group (Fig. S1 and S2). In the HSV 1/2 cohort, the <14-year age group had the highest proportion of cutaneous and oral lesions, while ages 20–29 had the highest proportion of genital lesions (Fig. S1). Within the VZV cohort, the <14 age group had the second highest proportion of total VZV positive individuals, with the highest proportion being from patients over the age of 70 (Fig. S2). Cutaneous VZV samples were more common in the <14 age group than in any other group, while the proportion of genital samples positive for VZV remained consistent across all age groups >14 (Fig. S2). Pediatric patients were included in this study to determine test performance in ages >19. For HSV-1, HSV-2, and VZV, the sensitivity, specificity, PPV, and NPV for pediatric patients <14 were 100% (Table S7). In the 14–19 age group, sensitivity, specificity, PPV, and NPV were 100% for HSV-1 and HSV-2, while sensitivity was 100%; specificity was 95.65%; PPV was 75.0%; and NPV was 100% for VZV in this age group (Table S7).

## DISCUSSION

This is the first report evaluating the clinical performance of the Savanna HSV 1+2/VZV assay compared to independent assays for HSV and VZV. These study data indicate that Savanna HSV 1+2/VZV assay results are comparable to those of Diasorin Simplexa HSV 1&2 and VZV direct kits.

Data from the current study are in alignment with those of two previous publications (7, 8), suggesting that positive and negative agreements and the ability of Savanna to detect across sample types and ages are consistent for this assay, regardless of the comparator method. Similar to previous studies on Savanna (7, 8), these data support the benefit of molecular multiplex testing for HSV and VZV in mucocutaneous and cutaneous lesions. The presence of HSV-1 and VZV identified in genital specimens supports previous studies (3, 5, 6, 9, 10). The prevalence of HSV in cutaneous (non-oral, non-genital) locations has not been well studied (8). However, this study found the incidence to be approximately 39% (30% HSV-1 and 9% HSV-2). The immunocompetency status of these patients is unknown, but future studies would benefit from collecting this level of patient information to determine the rates of immunocompromised status in patients with non-oral, non-genital HSV lesions.

Due to the lesion anatomical data provided by this study, it is important to note that both HSV and VZV lesions can be found in any anatomical location. This finding should be considered prior to therapy recommendations. Missed or misdiagnosed HSV and VZV infections have the potential to lead to unnecessary antibiotic use and prolonged symptoms that could be alleviated with proper antiviral treatment. These diagnostic errors also affect transmission control and isolation protocols, as VZV requires airborne precautions, while HSV needs contact precautions. How often these infections are missed, how factors like patient age or lesion location influence testing decisions, and how beneficial the use of a multiplexed HSV + VZV assay could be to clinical care remains poorly understood and an area for future research.

### Study limitations

While the evaluation of the Savanna HSV 1+2/VZV assay against other FDA-approved diagnostic platforms is essential for informing clinical implementation, several limitations must be acknowledged. These assays designed to assist in the differential detection of HSV-1, HSV-2, and VZV rely on nucleic acid amplification targeting viral DNA rather than viable organisms and, therefore, cannot confirm active infection. The study population was restricted to patients presenting with suspected HSV or VZV infections manifesting as cutaneous or mucocutaneous lesions, limiting generalization to atypical lesion presentations.

While all specimens with discordant results between Savanna and Simplexa were tested by a third assay (Solana), an equal number of concordant negative samples were not tested, which could result in a bias toward PPV. Thawed retrospective samples (*n* = 135) were also analyzed along with prospective data. However, freeze-thaw did not appear to affect assay efficacy, as only one specimen, which was ‘Simplexa-positive’ by clinical testing (HSV-2 positive, Ct = 39.3), went ‘Simplexa-negative’ after freeze-thaw, and this specimen was also negative when tested by Savanna. Discordant samples could also be affected by differences in the limit of detection (LoD) of each assay. Notably, mean Ct values reported by Simplexa and Savanna were statistically different, varying by 0.5 to 2.5 Ct for the three assay targets. Differences in Ct could impact LoD; however, other assay factors, including probe chemistry, genetic target, amplicon size, efficiency of heat transfer during cycling, etc., may also impact the Ct value reported. As such, the threshold for reporting a positive result may be different and not reflective of differences in LoD. Furthermore, the manufacturer claimed that the LoD as stated in the instructions for use for Savanna are as follows: HSV-1, 0.001–116 TCID_50_/mL; HSV-2, 8–25 TCID_50_/mL; and VZV, 1,002–3,320 copies/mL. Meanwhile, those for Simplexa are as follows: HSV-1, 4–160 TCID_50_/mL; HSV-2, 2–10 TCID_50_/mL; and VZV, 800–3,500 copies/mL. These suggest that discordance is most likely related to the specimen with a low viral concentration rather than substantial differences in LoD between the assays.

A potential benefit of multiplexed simultaneous testing for HSV and VZV is the identification of either virus in specimens with a clinical order only for HSV or VZV. While not the focus of this manuscript, a recent case report using the same Savanna assay directly addresses this question, and authors report up to 3.4% of “missed diagnosis” resulting from stand-alone HSV or VZV test orders (8). These were related to uncommon presentations as well as bias related to the location of lesion and patient age.

While this study found incidences that are previously unpublished, the study was not designed to address epidemiological biases. These findings may not be applicable to some populations,such as high risk. Further studies involving larger and more diverse sample sizes and expanded demographic data would enhance epidemiological insights. Moreover, longitudinal clinical follow-up is needed to assess whether multiplex assay results influence therapeutic decision-making.

### Conclusion

In summary, the findings of the current study show that the multiplex Savanna HSV 1+2/VZV assay is comparable to the individual Diasorin Simplexa HSV 1&2 and VZV direct assays. Introducing a multiplexed HSV + VZV assay would allow both viruses to be tested simultaneously from lesion swabs, minimizing dependence on clinical and epidemiologic judgment for test selection and enhancing diagnostic accuracy through laboratory confirmation.

## MATERIALS AND METHODS

### Study design

Three geographically distinct sites across the U.S. were included in this study. Sites were located in Wisconsin, Ohio, and New York. To clarify discordant cases, discrepant analysis was performed by a central laboratory (QuidelOrtho, Athens, OH). Study sites were reference and integrated health system laboratories that obtained samples from hospitals, emergency departments, and outpatient clinics. One site serviced both a pediatric hospital and an adult hospital.

This clinical study encompassed two primary cohorts to compare the performance of the Savanna HSV 1+2/VZV assay to that of Diasorin Simplexa HSV 1&2 Direct and Simplexa VZV Direct in cutaneous and mucocutaneous lesion swab specimens collected in transport media for routing clinical testing for suspected HSV-1, HSV-2, or VZV infections. Savanna HSV/VZV testing was conducted on remnant specimens submitted for clinical testing on the institutional tests of record, Simplexa HSV 1&2 and/or Simplexa VZV. Two sites obtained Institutional Review Board (IRB) approval from their institution, and one site obtained IRB approval from the Western IRB prior to commencement of the prospective field arm of this protocol. Informed consent was not required.

Basic demographic and medical history data, including sex, age, and clinical presentation, were recorded by study personnel for each enrolled patient. Enrollments spanned a 3-to-6-month period with extensions implemented as needed to obtain the protocol-specified minimum specimen numbers of both positive and negative cases.

### Clinical specimens

For the HSV-1/HSV-2 cohort, a total of 635 lesion swabs were enrolled over a 10-month period (April 2024–January 2025) from patients with signs and symptoms of HSV-1 or HSV-2 infection. Of the total, 499 specimens were tested fresh, comparing clinical Simplexa HSV 1&2 Direct test result to that for Savanna HSV 1+2/VZV testing of residual transport media specimen. The transport media types used for the swab study group included a universal transport medium (UTM; Copan [Murrieta, CA] and Hardy Diagnostics [Santa Maria, CA]), Avantik Viral Transport Medium (Avantik, Pine Brook, NJ), and Remel M4RT (Thermo Fisher Scientific, Waltham, MA). Samples were excluded if they did not meet the storage (2–8°C for up to 72 h post collection or at ≤−70°C thereafter) or volume (minimum 0.75 mL) requirements. For retrospective testing, 136 specimens were retained following clinical testing and stored frozen at ≤−70°C. To ensure equitable comparison following freeze-thaw, these retrospective specimens were thawed, tested on Savanna HSV 1+2/VZV, and re-tested by Simplexa HSV 1&2 Direct.

For the VZV cohort, a total of 374 lesion swabs were enrolled over a 13-month period (April 2024–May 2025) from patients with signs and symptoms of VZV infection. Of the total, 239 specimens were tested prospectively and 136 specimens tested retrospectively. Comparison testing of prospective and retrospective specimens by Savanna HSV 1+2/VZV and Simplexa VZV Direct was conducted with identical processes and exclusion criteria as described for the HSV1/HSV2 cohort.

Specimens that produced discrepant results were shipped frozen to QuidelOrtho laboratories for testing according to manufacturer protocols by Lyra Direct HSV 1+2/VZV real-time PCR assay (QuidelOrtho) and Solana HSV 1+2/VZV isothermal nucleic acid amplification assay (QuidelOrtho).

### Testing procedures

All assays included in this study were performed according to manufacturer protocols, including testing of external positive and negative controls in accordance with local laboratory policies. Study-specific training to ensure proper patient enrollment, specimen collection, testing, documentation, storage, and shipping of materials was conducted at all sites prior to protocol initiation.

### Statistical analysis

Positive percent agreement (PPA), negative percent agreement (NPA), overall percent agreement (OPA), and two-sided (upper/lower) 95% confidence interval were calculated for each cohort following discrepant analysis using Microsoft Office Excel 365 MSO software (Microsoft, Redmond, WA). The PPA was calculated as TP / (TP + FN) × 100; the NPA was calculated as TN / (TN + FP) × 100; and the OPA was calculated as (TP + TN) / *N* × 100, where TP is true-positive results; FN is false-negative results; TN is true-negative results; and FP is false-positive results. The dose-response 95th percentile (with 95% CI) model was assessed using the Finney and Stevens calculations. Cohen’s kappa values (*κ*) were also calculated as a measure of the OPA, with values categorized as almost-perfect (>0.90), strong (0.80 to 0.90), moderate (0.60 to 0.79), weak (0.40 to 0.59), minimal (0.21 to 0.39), or none (0 to 0.20).

## SUPPLEMENTAL MATERIAL

Supplemental material spectrum.03654-25-s0001.docx Patient demographics and Ct values.

ASM does not own the copyrights to Supplemental Material that may be linked to, or accessed through, an article. The authors have granted ASM a non-exclusive, world-wide license to publish the Supplemental Material files. Please contact the corresponding author directly for reuse.
