# Supplementary material for: Comparative evaluation of Savanna HSV 1+2/VZV multiplex assay and Simplexa HSV 1&2 and VZV direct kits for rapid molecular detection of HSV-1, HSV-2, and VZV
Source: Microbiol Spectr. 2026 Feb 4;14(3):e03654-25. doi: 10.1128/spectrum.03654-25 (PMC12955444; doi:10.1128/spectrum.03654-25)

Supplemental Table 1. Subject demographic characteristics.

| Specimen Source | Age | Female | Male | Total |
| --- | --- | --- | --- | --- |
| Cutaneous Lesion | < 14 | 62 | 61 | 123 |
|  | 14-19 | 22 | 21 | 43 |
|  | 20-29 | 28 | 22 | 50 |
|  | 30-39 | 28 | 12 | 40 |
|  | 40-49 | 32 | 19 | 51 |
|  | 50-59 | 27 | 12 | 39 |
|  | 60-69 | 39 | 27 | 66 |
|  | 70+ | 35 | 41 | 76 |
|  | Total | 273 | 215 | 488 |
| Mucocutaneous Lesion | < 14 | 31 | 43 | 74 |
|  | 14-19 | 51 | 12 | 63 |
|  | 20-29 | 92 | 22 | 114 |
|  | 30-39 | 57 | 11 | 68 |
|  | 40-49 | 33 | 14 | 47 |
|  | 50-59 | 42 | 12 | 54 |
|  | 60-69 | 31 | 10 | 41 |
|  | 70+ | 27 | 15 | 42 |
|  | Total | 364 | 139 | 503 |
| Total | < 14 | 93 | 104 | 197 |
|  | 14-19 | 73 | 33 | 106 |
|  | 20-29 | 125 | 46 | 171 |
|  | 30-39 | 86 | 23 | 109 |
|  | 40-49 | 65 | 34 | 99 |
|  | 50-59 | 71 | 24 | 95 |
|  | 60-69 | 72 | 37 | 109 |
|  | 70+ | 64 | 57 | 121 |
|  | Total | 649 | 358 | 1007 |

Supplemental Table 2. Comparison of positive and negative results for Savanna HSV 1+2/VZV Assay and Diasorin Simplexa HSV 1&2 Direct and VZV Direct testing and discrepant analysis by Lyra Direct HSV 1+2/VZV and Solana HSV 1+2/VZV.

|  | Savanna | Simplexa | |
| --- | --- | --- | --- |
|  |  | + | - |
| HSV-1 | + | 185 | 6 |
|  | - | 2 | 442 |
| HSV-2 | + | 157 | 1 |
|  | - | 0 | 477 |
| VZV | + | 126 | 1 |
|  | - | 0 | 247 |

HSV-1, herpes simplex virus 1; HSV-2, herpes simplex virus 2; VZV, varicella-zoster virus.

Supplemental Table 3. Discrepant specimens: HSV-1.

| Lesion Location | Lesion Type | Simplexa HSV-1 Result | Simplexa HSV-1  Ct | Savanna HSV-1 Result | Savanna HSV-1 Ct | Lyra HSV-1 Result | Lyra HSV-1 Ct | Solana HSV-1 Result |
| --- | --- | --- | --- | --- | --- | --- | --- | --- |
| Genital | Mucocutaneous | P | 38.5 | N | . | N | . | N |
| Oral | Mucocutaneous | P | 35.2 | N | . | P | 40 | P |
| Oral | Cutaneous | P | 39.8 | N | . | N | . | N |
| Oral | Mucocutaneous | P | 35 | N | - | N | - | P |
| Genital | Mucocutaneous | P | 37.9 | N | - | N | - | N |
| Oral | Cutaneous | P | 39.5 | N | - | N | - | N |
| Genital | Mucocutaneous | P | 36.4 | N | - | N | - | N |
| Genital | Mucocutaneous | P | 34.6 | N | - | N | - | N |
| Genital | Mucocutaneous | P | 37.9 | N | - | N | - | N |
| Cutaneous | Mucocutaneous | P | 38.9 | N | - | N | - | N |
| Cutaneous | Cutaneous | P | 35.8 | N | - | N | - | N |
| Cutaneous | Cutaneous | P | 39.6 | N | - | N | - | N |
| Oral | Cutaneous | P | 38.1 | N | - | N | - | N |
| Genital | Mucocutaneous | N | - | P | 33 | N | . | N |
| Genital | Mucocutaneous | N | - | P | 36 | N | . | N |
| Genital | Mucocutaneous | N | - | P | 40 | N | - | N |
| Oral | Mucocutaneous | N | - | P | 29 | N | - | N |
| Oral | Mucocutaneous | N | - | P | 37 | N | - | N |
| Genital | Mucocutaneous | N | - | P | 40 | N | - | N |

Supplemental Table 4. Discrepant specimens: HSV-2.

| Lesion Location | Lesion Type | Simplexa HSV-2 Result | Simplexa HSV-2  Ct | Savanna  HSV-2  Result | Savanna HSV-2  Ct | Lyra HSV-2 Result | Lyra HSV-2 Ct | Solana HSV-2 Result |
| --- | --- | --- | --- | --- | --- | --- | --- | --- |
| Genital | Mucocutaneous | P | 37.2 | N | . | N | . | N |
| Genital | Mucocutaneous | P | 40.4 | N | . | N | . | N |
| Genital | Mucocutaneous | P | 42 | N | - | N | - | N |
| Oral | Cutaneous | P | 40.4 | N | - | N | - | N |
| Cutaneous | Cutaneous | P | 39 | N | - | N | - | N |
| Genital | Mucocutaneous | P | 38.7 | N | - | N | . | N |
| Genital | Mucocutaneous | P | 38.8 | N | - | N | . | N |
| Genital | Mucocutaneous | N | - | P | 34 | N | . | N |

Supplement Table 5. Discrepant specimens: VZV.

| Lesion Location | Lesion Type | Simplexa VZV Result | Simplexa VZV Ct | Savanna VZV Result | Savanna VZV Ct | Lyra VZV Result | Lyra VZV Ct | Solana VZV Result |
| --- | --- | --- | --- | --- | --- | --- | --- | --- |
| Cutaneous | Cutaneous | P | 41.5 | N | - | N | - | N |
| Cutaneous | Cutaneous | P | 39.5 | N | - | N | - | N |
| Cutaneous | Cutaneous | P | 40.4 | N | - | N | - | N |
| Cutaneous | Cutaneous | N | - | P | 37 | N | - | N |

Supplement Table 6. Positive result Ct ranges for Savanna HSV 1+2/VZV Assay and Diasorin Simplexa HSV 1&2 Direct and VZV Direct testing.

|  | Simplexa | | | Savanna | | | *P* Value |
| --- | --- | --- | --- | --- | --- | --- | --- |
|  | Mean | Median | Range | Mean | Median | Range |  |
| HSV-1 | 25.3 | 24.9 | 16.1 - 38.6 | 24.8 | 24.0 | 14.0 - 40.0 | 0.0001 |
| HSV-2 | 26.9 | 26.4 | 15.3 - 37.6 | 28.5 | 28.0 | 17.0 - 40.0 | < 0.0001 |
| VZV | 26.3 | 25.1 | 18.8 - 39.2 | 22.6 | 21.0 | 14.0 - 38.0 | < 0.0001 |

Supplemental Table 7. Savanna HSV 1+2/VZV Assay clinical performance of pediatric samples by age category compared with Diasorin Simplexa HSV 1&2 Direct and VZV Direct testing and discrepant analysis by Lyra Direct HSV 1+2/VZV and Solana HSV 1+2/VZV.

|  |  | Total | TP | FN | TN | FP | Sensitivity %, | Specificity %, | PPV %, | NPV %, |
| --- | --- | --- | --- | --- | --- | --- | --- | --- | --- | --- |
|  |  |  |  |  |  |  | 95% CI | 95% CI | 95% CI | 95% CI |
| < 14 | HSV-1 | 128 | 59 | 0 | 69 | 0 | 100.00% | 100.00% | 100.00% | 100.00% |
|  |  |  |  |  |  |  | 93.94% to 100.00% | 94.79% to 100.00% | 93.94% to 100.00% | 94.79% to 100.00% |
|  | HSV-2 | 128 | 1 | 0 | 127 | 0 | 100.00% | 100.00% | 100.00% | 100.00% |
|  |  |  |  |  |  |  | 2.50% to 100.00%* | 97.14% to 100.00% | 2.50% to 100.00%* | 97.14% to 100.00% |
|  | VZV | 69 | 21 | 0 | 48 | 0 | 100.00% | 100.00% | 100.00% | 100.00% |
|  |  |  |  |  |  |  | 83.89% to 100.00% | 92.60% to 100.00% | 83.89% to 100.00% | 92.60% to 100.00% |
| 14-19 | HSV-1 | 80 | 20 | 0 | 60 | 0 | 100.00% | 100.00% | 100.00% | 100.00% |
|  |  |  |  |  |  |  | 83.16% to 100.00% | 94.04% to 100.00% | 83.16% to 100.00% | 94.04% to 100.00% |
|  | HSV-2 | 80 | 12 | 0 | 68 | 0 | 100.00% | 100.00% | 100.00% | 100.00% |
|  |  |  |  |  |  |  | 73.54% to 100.00% | 94.72% to 100.00% | 73.54% to 100.00% | 94.72% to 100.00% |
|  | VZV | 26 | 3 | 0 | 22 | 1 | 100.00% | 95.65% | 75.00% | 100.00% |
|  |  |  |  |  |  |  | 29.24% to 100.00%* | 78.05% to 99.89% | 30.61% to 95.33%* | 84.56% to 100.00% |

CI, confidence interval; FN, false negative; FP, false positive; HSV-1, herpes simplex virus 1; HSV-2, herpes simplex virus 2; NPV, negative predictive value; PPV, positive predictive value; TN, true negative; TP, true positive; VZV, varicella-zoster virus.
* Low sample size contributes to variation CI range.

Supplemental Figure 1. HSV 1/2 cohort lesion location by age group.
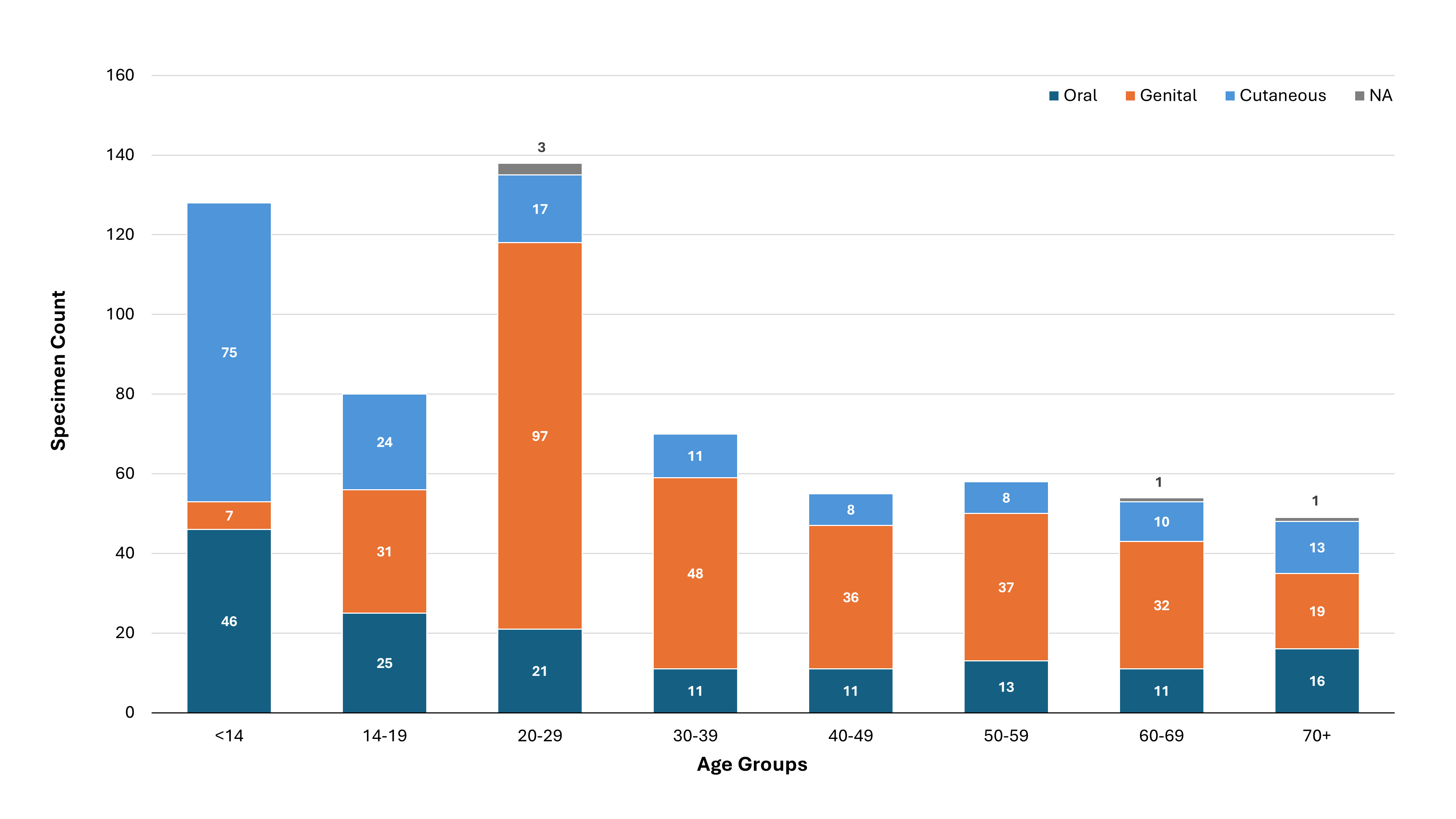


Supplemental Figure 2. VZV cohort lesion location by age group.
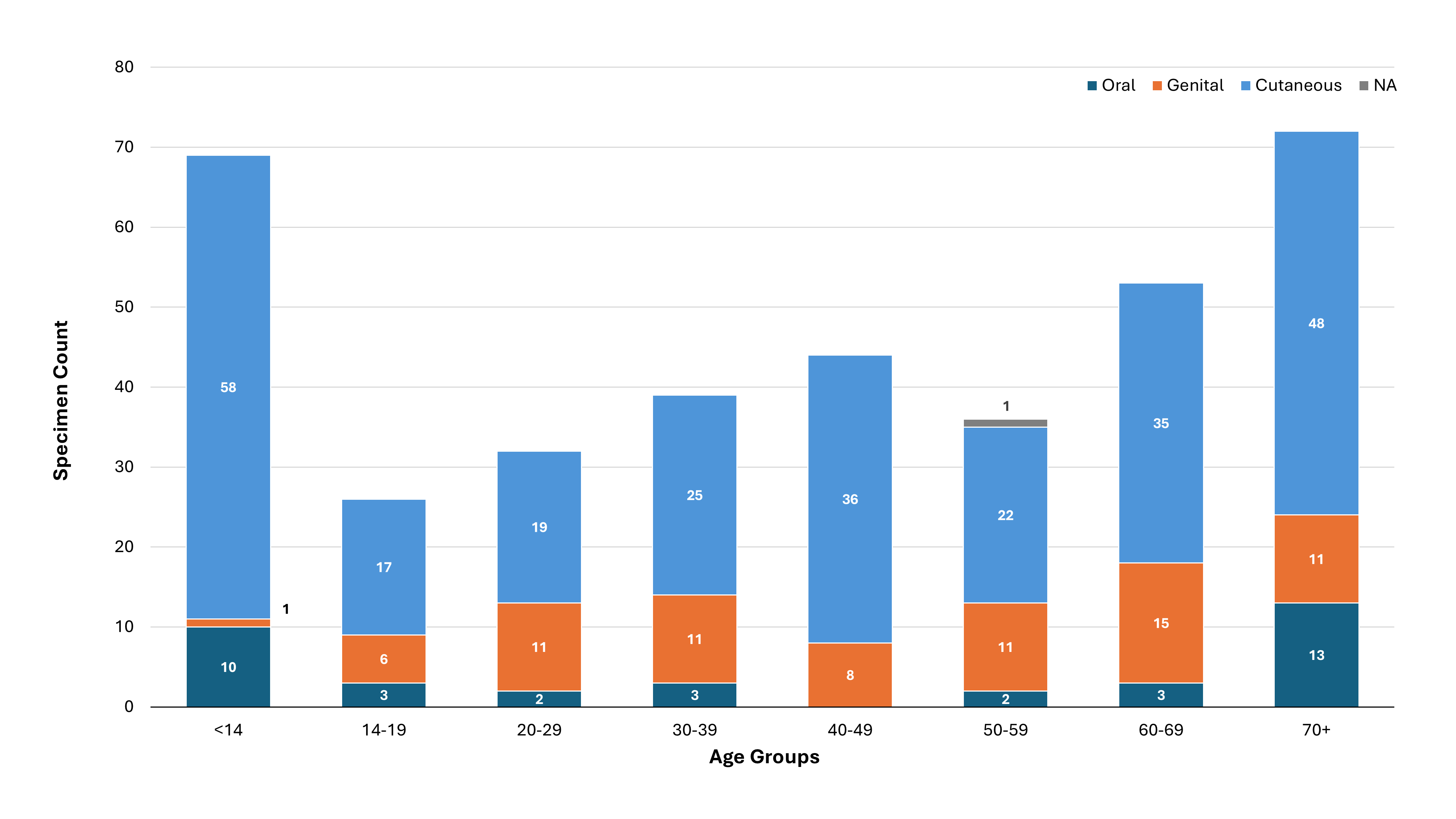

Supplement: Supplemental material — Patient demographics and Ct values. [file spectrum.03654-25-s0001.docx]
